# Circadian eating patterns track from infancy to pre- and primary school-age, but are not prospectively associated with body composition in childhood – Results of the DONALD cohort study

**DOI:** 10.1007/s00394-025-03673-2

**Published:** 2025-04-26

**Authors:** Ines Perrar, Eva Hohoff, Azadeh Lesani, Sarah Schmitting, Lars Libuda, Bettina Krueger, Bianca Stutz, Ute Nöthlings, Anette Buyken, Ute Alexy, Nicole Jankovic

**Affiliations:** 1 Institute of Nutritional and Food Sciences -Nutritional Epidemiology, University of Bonn, Friedrich-Hirzebruch- Allee 7, 53115 Bonn and Heinstück 11, 44225 Dortmund, Germany; 2 Institute of Nutrition, Consumption and Health, Faculty of Natural Sciences, Paderborn University, 33098 Paderborn, Germany; 3 Institute for Integrative Health Care and Health Promotion, Faculty of Health, Witten/Herdecke University, Witten, Germany

**Keywords:** Circadian pattern, Tracking, Children, Adolescents

## Abstract

**Background:**

While circadian eating patterns seem to be involved in the aetiology of obesity in adulthood. Little is known about tracking of such patterns from infancy to pre- and primary school age and their prospective impact on body composition.

**Methods:**

Based on data from the Dortmund Nutritional and Anthropometric Longitudinally Designed (DONALD) Study we investigated whether circadian eating patterns in infancy (0–1 years) (1) track to pre- (3–4 years) or primary-school age (6–7 years) and (2) are associated with body composition at primary-school age, using multivariable linear regression models. Circadian patterns were extracted from 3-day weighed dietary records (*n* = 3,780 records from *n* = 510 children) and related to body mass index standard deviation scores (BMI-SDS), fat mass index (FMI) and fat free mass index (FFMI).

**Results:**

Circadian eating patterns [i.e. eating occasion frequency (n/day), duration of nightly fasting (min), percentage of total energy intake from meals and snacks (%)] except carbohydrate intake after 6 p.m. track from infancy to pre- and primary school age (all *p* < 0.05). Sensitivity analyses stratified by breastfeeding duration (exclusive breastfed ≥ 4 months, yes/no) showed that the extent of tracking was less pronounced for ≥ 4 months breastfeeding. There were no significant associations between any aspect of circadian eating patterns in infancy and BMI-SDS or body composition during primary-school age (all *p* > 0.05).

**Conclusions:**

Our data suggest that circadian eating pattern in pre- and primary school age are shaped in infancy. However, this tracking of circadian eating pattern in childhood does not appear to have any health relevance with regard to BMI-SDS or body composition.

**Supplementary Information:**

The online version contains supplementary material available at 10.1007/s00394-025-03673-2.

## Background

Infancy is one of the critical phases regarding the risk for developing overweight and obesity later in life [1, 2]. So far, early life factors such as birth characteristics and breastfeeding status as well as the shaping of dietary patterns which establish in early childhood and track further into young childhood and adolescence are recognized for their importance on future weight as well as body compositional developments [3, 4].

Current research in Chrono-nutrition shows that not only the amount or type of food intake, i.e. “what we eat”, but also circadian eating patterns [5], i.e. “when we eat”, may be potential risk factors for the development of overweight and obesity [6]. Circadian eating patterns in childhood consider food consumption throughout the day in terms of a 24 - h cycle including all aspects of food intake, while the focus is on timing and regularity rather than what exactly is being consumed. As an example: circadian eating patterns may be characterized by e.g. irregular mealtimes or preferred consumption of foods or nutrients during specific times of the day (i.e. morning, day, evening [7, 8]. Our research group operationalized circadian eating patterns earlier for adolescence [8, 9] including the following variables which we apply for the first time in a group of infants and children in the current manuscript: diurnal meal timing (e.g. energy intake % from main meals or in between snacks, macronutrient intake in the morning or evening), eating occasion frequency (EOF defined as n/day) but also time restricted feeding (e.g. duration of nightly fasting (DNF)) [8].

There is much evidence regarding tracking of dietary patterns [10] and eating behaviour [11] such as food preferences, food variety [12], dietary intake [10, 13] or eating traits [14] from infancy to childhood. However, no such data exists for tracking of circadian eating from infancy to childhood. Also, there are no studies exploring the meaning of circadian eating patterns from infancy to early childhood and risk factors for future health. While i.e. diurnal meal timing or EOF are largely recognized in adults as a risk factor for metabolic regulation [15] and health [16] such associations remain unexplored during infancy and early childhood. Generally, we do have little knowledge when it comes to eating patterns and overweight development from infancy to childhood and beyond [17]. This is why we elaborate circadian eating patterns as defined above regarding tracking and longitudinal associations with body composition and body mass index (BMI).

To the best of our knowledge, only one longitudinal study investigated the association between eating frequency of meals and snacks at the age of two years with body mass index (BMI) z-scores at 1, 2 and 3.5 years of age among children in New Zealand [18], showing no significant results. So far, there is a lack of studies considering the stability of circadian eating patterns during infancy up to early school age and their influence on body compositional status. Therefore, aims of the present analyses were (1) to investigate tracking of circadian eating patterns between infancy (0–1 years) and pre- (3–4 years) or primary-school (6–7 years) age and (2) to examine the prospective associations between circadian eating patterns during infancy and BMI-standard deviation scores (BMI-SDS) as well as body composition (i.e. fat mass index (FMI) and fat free mass index (FFMI)) during primary-school age using data from the Dortmund Nutritional and Anthropometric Longitudinally Designed (DONALD) study.

## Methods

### Study population

The DONALD study is an ongoing, dynamic cohort study, which started in 1985 to collect data on diet, growth, development, and metabolism of children and adolescents in Dortmund, Germany and surrounding communities. About 35–40 infants are newly recruited every year. Eligible to participate are healthy infants (i.e., infants free of diseases affecting growth and/or dietary intake), whose parents are willing to participate in a long-term study and of whom at least one has sufficient knowledge of the German language. The participants are first examined at the age of three months and return for three more visits in the first year, two in the second year and thereafter annually until young adulthood. Regular examinations include among others 3-day weighed dietary records, anthropometric measurements and lifestyle interviews. Parental examinations (anthropometric measurements, lifestyle interviews) take place every four years. The study protocol and the basic data collection methods of the study were not changed during the entire study period. Further details about the study have been described elsewhere [19, 20]. The DONALD study was approved by the Ethics Committee of the University of Bonn (approval numbers: 098/06 and 185/20) according to the guidelines of the Declaration of Helsinki. All examinations are performed with parental consent and, from the age of 16 years onwards, on children’s written consent.

For the current analyses all participants with at least two 3-day weighed dietary records during infancy (0–1 year) as well as two records and anthropometric measurements each at pre-school age (3–4 years), and primary-school age (6–7 years) were included. This results in a study population of *n* = 510 participants (3,780 dietary records). Per participant, between two (*n* = 510, 100%) and four (*n* = 269, 52.5%) dietary records from infancy were available.

### Dietary assessment

Dietary data were collected using 3-day weighed dietary records. To estimate breast milk quantities during infancy, mothers were provided a baby scale (Soehnle multina 8300, Soehnle, Germany) to weigh the child before and after each feeding to the nearest 10 g [21, 22]. From the start of complementary feeding onwards, an electronic food scale (± 1 g) was used to weigh all foods and beverages consumed by the participant, as well as leftovers over three consecutive days. Participants chose the day of the beginning of dietary recording, usually within 8 weeks after the study centre visit. In addition to the consumed food, participants also document time and place of consumption.

After the three days of recording, a trained dietician crosschecks the dietary record for accuracy and completeness using standard questions (e.g. on brand, manufacturer, homemade recipes, preparation method, type) in a short interview/phone call with the participant. Thus, missing information can be added and it is inquired whether unusual events during the recording days might have changed the habitual dietary behaviour. The new collected dietary data is then added to our database. Subsequently, energy and nutrition intakes were calculated using our continuously updated in-house nutrient database LEBTAB [23]. The composition of staple foods in LEBTAB is based on the German food composition tables BLS 3.02 (https://www.blsdb.de/). Energy and nutrient contents of commercial food products, i.e., processed foods, ready-to-eat meals or snack foods are estimated by recipe simulation using labelled ingredients and nutrient contents. Total daily energy intake (TEI) and nutrient intakes were calculated as the individual mean of 3 days of recording.

### Circadian eating patterns

The selection of circadian eating pattern variables was driven by earlier research performed on this topic [9, 18, 24]. EOF [25], duration of nightly fasting [26], evening CHO intake [27] and the contribution of meals and snacks [28] have emerged from the literature as potential risk factors for excessive energy intake or overweight development. The following variables were defined according to standard procedures [24, 27] and based on consecutive 3-day weighed dietary records.

### Eating occasion frequency

Since there is no consensus regarding the time interval that defines beginning and end of one eating occasion we retained the same cut-off used in previous DONALD analyses [24, 29]. One eating occasion was defined as all foods and beverages consumed within a 30-min time-period. All eating occasions < 10 kcal were added to the previous eating occasion. Meals were defined as eating occasions providing > 10% of TEI (% TEI). Snacks were defined as eating occasions providing ≤ 10% TEI.

### Duration of nightly fasting

Start of the night was defined as the time of the day, after which < 5% of the last eating occasion was recorded and vice versa for the end of the night considering eating past 5 am. DNF (min) was defined as the longest time span with no energy intake (EI) ≥ 10 kcal) within one night. The individual mean of DNF obtained from the two nights per 3-day dietary record was then calculated. More details are described elsewhere [24].

### Morning and evening energy and macronutrient intake

Calculation of cut-points for morning (between age-specific wake-up time and 11 a.m.), daytime (from 11 a.m. to 6 p.m.) and evening (between 6 p.m. and the age-specific sleep) food intake during childhood and adolescence [30] is described in detail elsewhere [8, 24, 27, 30]. For the definition of the specific time windows, all dietary intakes were considered. The individual means of morning and evening energy or macronutrients intakes were calculated from the three record days.

For infancy, pre-school and primary school-age, the following parameters of circadian eating pattern were calculated based on data from 3 day weighed dietary records:

- EI from meals and snacks (in kcal and % of TEI); EI (kcal).
- morning, daytime and evening macronutrient intake (percent of EI (%EI)).
- frequency of nightly eating occasions (NEO, n/day).
- total EOF (n/day).
- total, morning, daytime and evening meal and snack frequency (n/day, respectively).

### Anthropometric data

Height and weight were measured by trained study nurses according to standard procedures with the participants dressed in underwear only and barefoot. Among children < 2 years of age, recumbent length is measured. From the age of 2 years onwards, standing height was measured to the nearest 0.1 cm using a digital stadiometer (Harpenden, Crymych, UK). Body weight is measured to the nearest 100 g using an infant weighing scale (until 08/2018: Mettler PS 15; Mettler Toledo, Columbus, OH; from 08/2018 onwards: Seca 757; Seca Weighing and Measuring System, Germany) as well as an electronic scale (Seca 920; from 02/2006 onwards Seca 701; Seca Weighing and Measuring System, Germany) among older participants. Body mass index [BMI (kg/m^2^ )] was calculated as the body weight (kg) divided by the square of the body height (m). BMI standard deviation scores (SDS, kg/m²) and overweight status (BMI-SDS > 90th percentile) were calculated using the German national reference data according to the LMS Method [31].

Triceps and subscapular skinfolds were measured on the right side of the body using a skinfold calliper (Holtain Ltd, Croswell, Dyfed, UK). The sum of both skinfolds was used for the estimation of percentage body fat according to the equations of Slaughter [32]. Absolute fat mass (body weight [kg] * percentage body fat) as well as fat free mass (body weight [kg] – body fat [kg]) were related to the square of height to obtain FMI (kg/m^2^) and FFMI (kg/m^2^).

All instruments are calibrated regularly. Quality controls of the measurements as well as coded data are also regularly conducted.

### Early life factors, family characteristics and physical activity

The child’s birth characteristics including birth weight were retrieved from a German standardized pregnancy document called “Mutterpass”. At the first study center visits the study pediatrician asks the mothers on how long (in weeks) they exclusive breastfed (no solid foods and no liquids other than breast milk). Additionally, the duration of any breastfeeding until weaning was assessed. Partial breastfeeding (breast milk additional to formula or complementary food) is calculated from duration of any breastfeeding minus duration of exclusive breastfeeding. For the German population a minimum of 4 months exclusive breastfeeding is advised [33] and served as the cut-off for the current analyses. Maternal body weight and height are measured with the same equipment as for the participants. Maternal overweight was defined as a BMI ≥ 25 kg/m^2^. High maternal educational status (≥ 12 years of schooling), maternal employment as well as smoking in the household were inquired using standardized questionnaires. For missing values of a specific covariate, the respective median of the total sample was used (*n* = 5 for maternal overweight during infancy, pre- and primary school age). Since 2004, a standardized questionnaire based on the Adolescent Physical Activity Recall Questionnaires [34] and questions from the German Health Interview and Examination Survey for Children and Adolescents (KiGGS) [35] is used to assess physical activity in the DONALD study starting at the age of three years. The metabolic equivalent in task hours per week (MET-h/week according to [36, 37]) was calculated for the present analyses.

### Statistical analyses

The statistical analyses of the present evaluation were performed using SAS^®^ procedures (version 9.4; Cary, NC, USA). The significance level was set at *p* < 0.05. Descriptive analyses were conducted and presented as medians with 25th and 75th percentiles for continuous variables or as frequencies and percentages for categorical variables (Table 1 and Additional file 1).

Multivariable linear regression models were used to investigate tracking of circadian eating pattern variables i.e., EOF (n/day), DNF (min), energy from meals (% of TEI) and from snacks (% of TEI), carbohydrate intake [%EI] after 6 p.m. between infancy and pre- or primary-school age. Tracking of NEO (n/d) was not analysed, since dietary records of pre- and primary school aged participants did not show any NEO (Table 1, Additional file 2). For the analyses, data from multiple dietary records were averaged i.e. at least two in each age group. The respective eating pattern variables in infancy was chosen as predictor, the corresponding variable in pre- or primary-school age as outcome. To limit the possibility of false positive results due to the high number of statistical analyses, we controlled for multiple testing by holding the False Discovery Rate (FDR) at 5% [38]. Prospective associations between circadian pattern during infancy and body composition (BMI-SDS, FMI, FFMI) during primary-school age were analysed by multivariable linear regression. The decision for the inclusion of covariates based on a previous DONALD analysis ( [39]).

Since breastfeeding appears to be a relevant confounder or effect modifier: as it is discussed as an important risk factor for the development of obesity [1], first interactions between predictor variables and sex as well as breastfeeding duration (exclusive breastfed ≥ 4 months vs. exclusive breastfed < 4 months) were tested. Testing for interaction did not reveal significant interaction. Nevertheless, sensitivity analyses were performed, including the same analyses with a sample stratified by breastfeeding duration to assess potential weaker associations in one group or the other (Additional file 2–6).

All regression models were tested for linearity, heteroskedasticity, multicollinearity and normal distribution of residuals.

Since standardized data on physical activity was collected since 2004 only, a sensitivity analyses for the prospective associations between circadian pattern during infancy and body composition (BMI-SDS, FMI, FFMI) during primary-school were performed. For this purpose, only participants for whom physical activity data at primary-school age were available (*n* = 303) were examined and MET-h/week was included in the models as an additional confounder. However, the sensitivity analyses led to the same results as the main analysis (see additional file 5).

## Results

Of the *N* = 510 participants included *n* = 243 (47.7%) were boys. Detailed circadian, anthropometric and socioeconomic characteristics of infancy, preschool and primary school age are shown in Table 1. Median circadian eating patterns of the total sample differed only slightly during the course of childhood. However, median NEO decrease after infancy, while DNF increases. The study population was characterized by a high maternal educational status (> 75%) and had a low prevalence of overweight children during primary school age (9.7%). The high socio-economic status is also reflected in the breastfeeding rates in the study. Most participants were exclusive breastfed ≥ 4 months (69.0%, Additional file 1). Characteristics stratified by breastfeeding duration, are shown in the additional files (Additional file 2). The respective median values indicate that circadian eating patterns differed by breastfeeding duration during infancy regarding total daily energy intake and macronutrient distribution during the day but not in terms of the distribution in different time windows. In addition, infants who were breastfed ≥ 4 months had slightly larger median EOF during infancy, due to higher median NEO and a shorter median DNF.

Multivariable regression models showed that all investigated variables besides evening carbohydrate intake (after 6 p. m.) track significantly from infancy to pre-school and primary-school age (Table 2). However, the extent of tracking seems to diminish with increasing age. No prospective associations between circadian eating patterns in infancy and BMI-SDS, FMI and FFMI in primary-school age were observed (Table 3).

**Table 1:** Chronobiological, dietary, anthropometric and socioeconomic characteristic from 510 DONALD participants during infancy (0–1 year), pre-school (3–4 years) and primary school age (6–7 years)

	Infancy	Pre-school Age	Primary School Age
**Age** [years]	0.7 (0.6; 0.8)	3.5 (3.5; 3.6)	6.5 (6.5; 6.6)
**Total daily energy intake (EI) [kcal]**	689 (628; 746)	1130 (1029; 1253)	1456 (1313; 1611)
from meals [%]	93.2 (88.8; 96.2)	90.1 (85.6; 93.6)	91.7 (88.4; 94.4)
from snack [%]	9.3 (5.6; 13.1)	11.6 (8.5; 15.3)	10.2 (7.7; 13.3)
**Total daily macronutrient intake [%EI]**			
Carbohydrate intake	49.1 (46.5; 52.3)	52.4 (48.6; 55.7)	52.5 (49.4; 56.0)
Fat intake	39.3 (35.8; 42.5)	33.7 (30.2; 37.0)	33.8 (30.7; 36.7)
Protein intake	10.3 (9.3; 11.5)	12.9 (11.7; 14.1)	12.5 (11.5; 13.8)
**Morning EI and Macronutrient intake**			
EI [kcal]	192 (165; 225)	343 (288; 409)	645 (554; 749)
Carbohydrate intake [%EI]	47.0 (43.6; 50.4)	53.5 (48.1; 59.3)	53.8 (49.8; 58.1)
Fat intake [%EI]	42.2 (39.1; 46.2)	32.2 (26.9; 37.4)	33.2 (29.3; 36.8)
Protein intake [%EI]	9.4 (8.2; 10.9)	12.9 (11.3; 14.7)	11.7 (10.3; 13.4)
**Daytime EI and Macronutrient intake**			
EI [kcal]	268 (235; 306)	510 (440; 585)	411 (378; 493)
Carbohydrate intake [%EI]	51.5 (47.8; 55.0)	55.0 (50.2; 59.5)	55.0 (50.0; 59.4)
Fat intake [%EI]	36.9 (32.9; 41.0)	31.7 (28.1; 36.1)	31.9 (27.0; 36.0)
Protein intake [%EI]	10.3 (9.2; 11.8)	11.6 (10.5; 13.3)	12.2 (10.7; 14.0)
**Evening EI and Macronutrient intake**			
EI [kcal]	186 (158; 218)	264 (212; 313)	363 (300; 443)
Carbohydrate intake [%EI]	48.1 (45.2; 52.3)	46.9 (40.1; 53.1)	48.0 (42.5; 53.7)
Fat intake [%EI]	39.5 (35.1; 43.3)	37.3 (31.0; 42.8)	36.5 (31.5; 41.3)
Protein intake [%EI]	10.9 (9.3; 12.6)	14.6 (12.5; 16.6)	14.1 (12.4; 15.9)
**Eating frequencies** **Whole day**			
Eating occasion frequency (n/day)	5.7 (5.1; 6.6)	6.0 (5.2; 6.7)	5.5 (5.0; 6.2)
Meal frequency (n/day)	4.5 (4.2; 4.8)	4.0 (3.9; 4.3)	4.0 (3.7; 4.2)
Snack frequency (n/day)	1.2 (0.7; 1.8)	1.7 (1.2; 2.7)	1.5 (1.0; 2.2)
**Morning**			
Eating occasion frequency (n/day)	1.8 (1.5; 2.0)	1.8 (1.7; 2.2)	1.7 (1.3; 1.8)
Meal frequency (n/day)	1.3 (1.1; 1.5)	1.3 (1.2; 1.5)	1.2 (1.0; 1.3)
Snack frequency (n/day)	0.4 (0.2; 0.7)	0.5 (0.3; 0.8)	0.3 (0.2;0.5)
**Daytime**			
Eating occasion frequency (n/day)	2.3 (2.1; 2.5)	2.8 (2.3; 3.3)	2.7 (2.3; 3.2)
Meal frequency (n/day)	1.8 (1.7; 2.0)	1.8 (1.5; 2.0)	1.7 (1.5, 2.0)
Snack frequency (n/day)	0.4 (0.3; 0.7)	1.0 (0.5; 1.5)	0.8 (0.5; 1.3)
**Evening**			
Eating occasion frequency (n/day)	1.3 (1.1; 1.7)	1.2 (1.0; 1.3)	1.2 (1.0; 1.3)
Meal frequency (n/day)	1.1 (1.0; 1.3)	1.0 (0.8; 1.0)	1.0 (0.8; 1.0)
Snack frequency (n/day)	0.2 (0.1; 0.4)	0.2 (0.0; 0.3)	0.2 (0.0; 0.3)
**Nightly eating occasions (n/day)**	0.6 (0.3; 0.8)	0.0 (0.0; 0.0)	0.0 (0.0; 0.0)
**Duration of nightly fasting (min)**	624 (524; 709)	795 (761; 831)	800 (768; 836)
**Anthropometric data**			
Body Weight (kg)	8.4 (7.7; 9.1)	16.0 (14.8; 17.3)	22.8 (21.1; 25.4)
BMI-SDS	0.57 (-0.05; 1.22)	0.07 (-0.43; 0.63)	-0.11 (-0.59; 0.51)
Overweight [%]^1^	160 (31.4)	51 (10.0)	47 (9.2)
FMI	2.96 (2.52; 3.43)	2.40 (2.01; 2.84)	2.20 (1.81; 2.71)
FFMI	14.11 (13.57; 14.66)	13.2 (12.7; 13.7)	13.2 (12.7; 13.8)
**Socioeconomic factors [%]**			
Maternal overweight^2^	156 (30.6)	160 (31.3)	180 (35.3)
Maternal high educational status^3^	386 (75.7)	385 (75.5)	392 (76.9)
Maternal employment	95 (18.6)	231 (45.3)	365 (71.6)
Smoking in Household^4^	77 (17.7)	70 (16.1)	71 (16.3)

Values are frequencies (n (%)) or medians (25th; 75th percentile)

EI, energy intake; %EI = percentage of energy intake, BMI, body mass index

^1^Overweight status (BMI-SDS > 90th percentile) was calculated based on the German reference percentiles for children and adolescents by Kromeyer-Hauschild [31] ^2^BMI > 25 kg/m²;

^3^≥12 years of schooling; ^4^*n*=74 missings

**Table 2:** Prospective associations between circadian eating patterns in infancy (0–1 years) with respective eating patterns in pre-school (3–4 years) or primary-school (6–7 years) age of the analysed DONALD sample

	**Association between infancy and pre-school age (3–4 years)**			
	Infancy (0–1 years)	Pre-school age (3–4 years)	β	P_(FDR)_^1^
**Circadian eating pattern**	Median (25th; 75th percentile)	Median (25th; 75th percentile)		
**Eating occasion frequency (n/day)**	5.7 (5.1; 6.6)	6.0 (5.2; 6.7)	0.24	**< 0.01**
**Duration of nightly fasting (min)**	624 (524; 709)	795 (761; 831)	0.11	**< 0.01**
**Energy from meals (% of TEI)**	93.2 (88.8; 96.2)	90.1 (85.6; 93.6)	0.15	**< 0.01**
**Energy from snacks (% of TEI)**	9.3 (5.6; 13.1)	11.6 (8.5; 15.3)	0.12	**< 0.01**
**Evening carbohydrate intake [%EI]**	48.1 (45.2; 52.2)	46.9 (40.1; 53.1)	-0.04	0.71
	**Association between infancy and primary-school age (6–7 years)**			
	**Infancy (0–1 years)**	**Primary-school age (6–7 years)**	**β**	**P**_**(FDR)**_^**1**^
**Circadian eating pattern**	Median (25th; 75th percentile)	Median (25th; 75th percentile)		
**Eating occasion frequency (n/day)**	5.7 (5.1; 6.6)	5.5 (5.0; 6.2)	0.15	**< 0.01**
**Duration of nightly fasting (min)**	624 (524; 709)	800 (768; 836)	0.05	**< 0.05**
**Energy from meals (% of TEI)**	93.2 (88.8; 96.2)	91.7 (88.4; 94.4)	0.08	**< 0.05**
**Energy from snacks (% of TEI)**	9.3 (5.6; 13.1)	10.2 (7.7; 13.3)	0.09	**< 0.05**
**Evening carbohydrate intake [%EI]**	48.1 (45.2; 52.2)	48.0 (42.4; 53.7)	0.00	0.99

EI = energy intake; TEI = Total daily energy intake; FDR = False discovery rate

^1^P_(FDR)_ refers to P values obtained in linear regression models. Models are adjusted for sex, exclusive breastfeeding over 4 month (yes/no), date of birth, birth weight, maternal high educational status during infancy, maternal employment and weight status during primary-school age. Multiple testing adjustments were performed using the false discovery rate at 5%

**Table 3:** Prospective associations between circadian eating patterns in infancy with BMI-SDS/FMI/FFMI in primary-school (6–7 years) age of the analysed DONALD sample

	BMI-SDS		FMI		FFMI	
**Circadian pattern in infancy**	**β**	P_value_	**β**	P_value_	**β**	P_value_
**Eating occasion frequency (n/day)** Unadjusted model Full adjusted model	-0.0180 0.0265	0.54 0.38	-0.0175 0.0490	0.59 0.14	-0.0046 0.0152	0.88 0.63
**Duration of nightly fasting (min)** Unadjusted model Full adjusted model	0.0004 -0.0001	0.11 0.61	0.0005 -0.0002	0.08 0.51	0.0002 -0.0002	0.44 0.61
**Energy from meals (% of TEI)** Unadjusted model Full adjusted model	0.0023 -0.0049	0.63 0.32	0.0015 -0.0080	0.78 0.14	0.0016 -0.0027	0.75 0.60
**Energy from snacks (% of TEI)** Unadjusted model Full adjusted model	-0.0038 0.0040	0.45 0.44	-0.0021 0.0078	0.70 0.16	-0.0035 -0.0014	0.50 0.80
**Carbohydrate intake [%EI] after 6 p.m.** Unadjusted model Full adjusted model	0.0042 -0.0043	0.54 0.52	0.0041 -0.0009	0.58 0.91	0.0076 -0.0037	0.29 0.59

BMI = body mass index, FMI = Fat mass index, FFMI =  Fat free mass index; TEI = Total daily energy intake

Fully adjusted model adjusted for sex, exclusive breastfeeding > 4 month, birthdate, birth weight, maternal high educational status during infancy, maternal employment and weight status during pre- or primary-school age and for total daily energy intake during infancy

Sensitivity analyses show the tracking of circadian patterns, except evening carbohydrate intake, from infancy to preschool age also in the stratified sample. For children who were breastfed ≥ 4 months, we only observed an association between EOF in infancy and the EOF at primary- school age (β = 0.12, p _(FDR)_ = < 0.01; Additional file 3). Among children who were breastfed < 4 months, the same significant associations were observed as in the total sample, with the exception of DNF. In line with the main analyses, no associations were observed between the circadian patterns in infancy and BMI-SDS, FMI and FFMI in primary-school age among the stratified sample (Additional files 4).

## Discussion

To our knowledge, this is the first study presenting (1) data on tracking of circadian eating patterns from infancy to pre- and primary-school age and investigating (2) the association of these circadian patterns with body composition in later childhood. Our results suggest that shaping of circadian eating patterns already starts during infancy. However, there is no long-term association between infant circadian eating patterns and BMI-SDS or body composition during childhood, which may be related to a disproportionately long exclusive BF duration as observed for the current sample and the inclusion of children up to age 7 and not longer.

There is a lack of knowledge regarding the stability of circadian eating patterns in early childhood especially starting from infancy. Previous studies that investigated the behavior of European children mainly showed existing tracking of dietary patterns i.e. the combination of food group or nutrient intakes from infancy up to early childhood (age 2–8 years) [10, 40]. Circadian eating patterns during childhood also appear to be rather stable even at “critical” time points like adolescence with the exception of the circadian distribution of energy and macronutrient intake [8, 27]. Considering, age and time trends of circadian eating patterns, i.e., EOF and DNF as well as TEI from meals and snacks derived earlier in the DONALD study, appeared to be fairly stable over the study period from childhood up to adolescence [24]. In accordance to the present DONALD data, also a large European-wide study revealed, EOF to be rather stable across ages from 3 (5.7 EOF/day) to 8 years (5.1 EOF/day) [41]. There is no specific work on tracking of TEI of meals and snacks but it was shown earlier that snacking behavior establishes in early childhood and show first diverging patterns in comparison to childhood during adolescence at the earliest [11]. Girls appeared to be more prone for changes in snacking patterns due to body weight concerns. As a consequence, eating patterns may change more substantially during the transition from later childhood to adolescence, but appear to be rather stable from infancy up to early childhood, which is in accordance with changes reported earlier for dietary patterns [42].

Current advice to maintain child health does not consider circadian eating patterns due to the lack of evidence [43]. Although, the impact of circadian intake on later risk development is highly recognized at older age [6, 16] . To the best of our knowledge previous studies included the age of 3 years onwards and assessed BMI or body composition prospectively. A recent approach was performed by Jaeger et al. [44] who assessed circadian distribution of macronutrient intake on BMI z-scores from age 3 up to age 8 years. Like our results, no significant associations were observed. Earlier results of the DONALD study reported high carbohydrate intake and low fat intake in the morning at age 3/4 and 7/8 years were associated with higher FMI values during adolescence. The interpretation of this endpoint is somewhat difficult since also FFMI values increased but did not reach significance [27]. Hence, more research is necessary regarding the interpretability of results especially in this early life stage. There are a few other studies showing homogeneous results regarding diurnal distribution of macronutrients [45] or energy intake [45, 46] and BMI or body composition. Considering our results in the light of previous findings it seems that infant circadian eating patterns are unrelated to future body compositional developments up until school age under the assumption that infants are supported in their individual feeding rhythm [47].

Our results stratified by breastfeeding duration revealed no clear difference in both groups regarding the association with later body compositional outcomes. However, we observed slightly weaker associations for the tracking of circadian eating patterns among children who were exclusive breastfed ≥ 4 month compared to participants with a shorter breastfeeding duration. This may indicate greater flexibility among breastfed children towards pattern changes in the future [11].

The main strength of this study is its longitudinal design, allowing to investigate tracking of circadian patterns from infancy to childhood. A further strength of the study are the repeated weighed dietary records and detailed day-time specific nutritional data for every individual included in the analysis. A minimum of two dietary records were used for the current analyses which is of great importance since the punctual assessment of food timing is insufficient [48] and likely results in erroneous associations and may also cause differences in associations in comparison to previous studies [45]. Due to the comprehensive study design, several factors (birth weight, socioeconomic factors, maternal overweight, physical activity) which are discussed in relation to the development of overweight [1–3] could already be taken into account as covariates. However, the development of obesity is very complex and multi-dimensional [1–3], so that other factors (e.g. metabolic or dietary factors, genetics) but also transitions related to age, like influences by peer-groups [49] and changes in chronotype [50]), could play a role, but could not be considered in the present analyses. As in the present analysis, the focus is on circadian factors, and although a number of covariates has been included in the analysis, risk of bias by residual confounding cannot be excluded. Future studies may enhance the follow-up period to adolescence or if possible young adulthood, to observe associations between circadian eating patterns and above-mentioned transitions occurring later in life.

A limitation regarding comparability with other studies is the use of self-defined cut-offs for eating time and hence morning or evening intakes. An additional measure for EO would benefit the analyses, enable verification of the exposure definition and enhance comparability across studies. The DONALD sample suffers from the same drawbacks as all other cohort studies in sampling mostly very well-situated families as can be seen by the high level of SES (Table 1). Furthermore, in our DONALD sample a high number of women exclusive breastfed for at least 4 months (about 70%). The length of exclusive breastfeeding of about 5 months and total breastfeeding of almost 9 months in DONALD shows a rather high commitment to the adherence of breastfeeding guidelines [33] which is not in accordance with the KIGGS Study, a representative cohort study of German children [51]. Here, only 40% of all children were exclusive breastfed for 4 months [52]. Again, high SES may benefit potential short comings that could occur in children receiving shorter breastfeeding [53]. Hence, the observed associations stratified by breastfeeding duration may be diluted in the current sample. The unexpected similarity in longer vs. shorter breastfeeding could also be influenced by weaning practices that we did not take into account in the current analyses [11]. Standardized physical activity assessments were implemented since 2004 in DONALD [20] and start at the age of three. Hence, we could not include baseline physical activity in our regression analyses due to missing data. However, since infancy was the baseline and physical activity to that time may be intrinsically motivated in healthy children, the level of confounding may be neglectable. In addition, we conducted sensitivity analyses including participants for whom data on physical activity at primary- school age was available. These led to the same conclusions as the main analyses.

## Conclusion

To conclude, circadian eating patterns track from infancy to early childhood. There was no prospective association between circadian eating patterns during infancy and BMI-SDS or body composition at age 7–8. Tracking for longer breastfed children was less pronounced in comparison to shorter breastfeeding but associations with body composition in childhood were comparable in both groups.

## Electronic supplementary material

Below is the link to the electronic supplementary material.

Supplementary Material 1

## Data Availability

Data of the DONALD study is available upon request to epi@uni-bonn.de.

## Abbreviations

BMI: Body mass index
BMI-SDS: Body mass index standard deviation scores
DNF: Duration of nightly fasting
DONALD: Dortmund Nutritional and Anthropometric Longitudinally Designed
EOF: Eating occasion frequency
FDR: False discovery rate
FMI: Fat mass index
FFMI: Fat free mass index
NEO: Nightly eating occasion
TEI: Total energy intake
