# Supplementary material for: Circadian eating patterns track from infancy to pre- and primary school-age, but are not prospectively associated with body composition in childhood – Results of the DONALD cohort study
Source: Eur J Nutr. 2025 Apr 26;64(4):165. doi: 10.1007/s00394-025-03673-2 (PMC12033188; doi:10.1007/s00394-025-03673-2)
Supplement: Supplementary file 1 — Supplementary Material 1 [file 394_2025_3673_MOESM1_ESM.docx]

**Additional files**

**Additional file 1:** Early life characteristics of the analysed DONALD sample (n=510)

| **Females** [n (%)] | 243 (47.7)/ |
| --- | --- |
| **Early life characteristics** |  |
| Year of birth [year] | 1998 (1991; 2004) |
| Birth weight [g] | 3400 (3120; 3770) |
| Gestational age [week] | 40 (39; 41) |
| Gestational weight gain [kg] | 14 (11; 16.3) |
| Exclusive breastfeeding ≥ 4 months [n (%)] | 352 (69.0) |
| Exclusive breastfeeding in weeks | 21 (8; 26) |
| Total breastfeeding in weeks^1^ | 34 (19; 46) |

Values are frequencies (n (%)) or medians (25th; 75th percentile)

^1^Data available from only 484 participants

**Additional file 2.** Circadian, dietary, antropometric and socioecomonic characteristic from 510 DONALD participants during infancy (0-1 year), pre-school (3-4 years) and primary school age (6-7 years), , stratified by breastfeeding duration (exclusive breastfed ≥ 4 month (n=352) vs. exclusive breastfed < 4 month (n=158))

|  | **Infancy** | | **Pre-school Age** | | **Primary School Age** | |
| --- | --- | --- | --- | --- | --- | --- |
|  | **Exclusive Breastfed ≥ 4 months** | **Exclusive Breastfed ≤ 4 months** | **Exclusive Breastfed ≥ 4 months** | **Exclusive Breastfed ≤ 4 months** | **Exclusive Breastfed ≥ 4 months** | **Exclusive Breastfed ≤ 4 months** |
| **n** | 352 | 158 | 352 | 158 | 352 | 158 |
| **Age** [years] | 0.7 (0.6; 0.8) | 0.7 (0.6; 0.8) | 3.5 (3.5; 3.6) | 3.5 (3.5; 3.6) | 6.5 (6.5; 6.6) | 6.5 (6.5; 6.6) |
| **Total daily energy intake (EI) [kcal]** | 677 (620; 735) | 715 (655; 772) | 1117 (1032; 1237) | 1153 (1023; 1284) | 1446 (1314; 1605) | 1482 (1311; 1623) |
| from meals [%] | 91.8 (86.4; 95.4) | 95.1 (92.0; 97.2) | 89.7 (85.6; 92.9) | 90.6 (86.2; 94.3) | 91.6 (88.5; 94.4) | 92.0 (88.3; 94.6) |
| from snack [%] | 10.2 (6.8; 14.6) | 7.0 (4.5; 10.2) | 11.8 (8.6; 15.6) | 11.2 (8.0; 14.8) | 10.2 (7.7; 13.4) | 9.9 (7.6; 13.3) |
| **Total daily macronutrient intake [%E]** |  |  |  |  |  |  |
| Carbohydrate intake | 48.2 (45.8; 51.5) | 50.3 (48.2; 53.2) | 52.4 (48.7; 55.5) | 52.5 (48.3; 55.9) | 52.5 (49.6; 56.0) | 52.3 (48.8; 56.1) |
| Fat intake | 40.8 (36.7; 43.7) | 37.5 (34.6; 39.5) | 33.7 (30.2; 37.0) | 33.4 (30.0; 36.9) | 33.9 (30.9; 36.7) | 33.6 (30.5; 36.9) |
| Protein intake | 10.0 (8.9; 11.1) | 11.0 (10.1; 12.1) | 12.9 (11.7; 14.1) | 13.0 (11.7; 14.0) | 12.4 (11.4; 13.7) | 12.9 (11.7, 13.9) |
| **Morning EI and Macronutrient intake** |  |  |  |  |  |  |
| EI [kcal] | 188 (159; 218) | 207 (174; 239) | 342 (287; 407) | 345 (291; 412) | 409 (336; 493) | 412 (248; 491) |
| Carbohydrate intake [%E] | 46.0 (43.2; 49.8) | 48.3 (45.4; 51.4) | 53.0 (47.6; 59.2) | 53.9 (49.9; 59.4) | 54.9 (49.8; 59.2) | 55.5 (50.4; 59.8) |
| Fat intake [%E] | 43.4 (39.9; 47.0) | 40.2 (37.3; 42.7) | 32.4 (27.1; 37.6) | 31.7 (26.5; 36.3) | 32.2 (27.2; 36.3) | 31.4 (26.5; 35.6) |
| Protein intake [%E] | 8.9 (8.0; 10.5) | 10.2 (9.4; 11.4) | 13.0 (11.3; 14.8) | 12.7 (11.3; 14.7) | 12.1 (10.7; 13.8) | 12.4 (10.9; 14.2) |
| **Daytime EI and Macronutrient intake** |  |  |  |  |  |  |
| EI [kcal] | 262 (229; 302) | 285 (245; 317) | 506 (439; 579) | 516 (445; 606) | 649 (553; 757) | 632 (558; 742) |
| Carbohydrate intake [%E] | 50.7 (46.6; 54.8) | 53.0 (50.0; 55.6) | 54.6 (50.5; 59.2) | 55.2 (49.9; 59.9) | 53.8 (49.8; 57.9) | 53.5 (49.7; 58.2) |
| Fat intake [%E] | 38.0 (33.7; 42.8) | 35.2 (31.9, 37.8) | 32.1 (28.1; 36.0) | 31.1 (27.4; 36.5) | 33.3 (29.3; 36.9) | 32.8 (29.0; 36.7) |
| Protein intake [%E] | 10.0 (8.9; 11.4) | 11.0 (10.0; 12.5) | 11.6 (10.2; 13.3) | 11.9 (10.6; 13.5) | 11.7 (10.3; 13.1) | 11.8 (10.3; 13.6) |
| **Evening EI and Macronutrient intake** |  |  |  |  |  |  |
| EI [kcal] | 180 (153; 210) | 202 (170; 225) | 260 (207; 306) | 278 (219; 321) | 358 (296; 431) | 370 (306; 462) |
| Carbohydrate intake [%E] | 47.4 (44.6; 51.4) | 49.4 (46.6; 53.8) | 47.0 (40.0; 53.1) | 46.3 (40.6; 52.4) | 47.8 (42.6; 53.6) | 48.4 (42.0; 53.9) |
| Fat intake [%E] | 40.7 (36.0; 43.8) | 37.1 (33.7; 41.1) | 37.3 (30.8; 42.8) | 37.3 (31.4; 42.7) | 36.7 (31.4; 41.4) | 36.1 (31.5; 41.1) |
| Protein intake [%E] | 10.4 (8.8; 12.3) | 11.5 (10.2; 13.1) | 14.6 (12.6; 16.6) | 14.6 (12.5; 16.5) | 14.1 (12.0; 15.9) | 14.1 (12.9; 16.1) |
|  |  |  |  |  |  |  |

Continuation on next page

**Continuation Additional file 2.** Chronobiological, dietary, antropometric and socioecomonic characteristic from 510 DONALD participants during infancy (0-1 year), pre-school (3-4 years) and primary school age (6-7 years), stratified by breastfeeding duration (exclusive breastfed ≥ 4 month (n=352) vs. exclusive breastfed < 4 month (n=158))

|  | **Infancy** | | **Pre-school Age** | | **Primary School Age** | |
| --- | --- | --- | --- | --- | --- | --- |
|  | **Exclusive Breastfed ≥ 4 months** | **Exclusive Breastfed ≤ 4 months** | **Exclusive Breastfed ≥ 4 months** | **Exclusive Breastfed ≤ 4 months** | **Exclusive Breastfed ≥ 4 months** | **Exclusive Breastfed ≤ 4 months** |
| **Eating frequencies**  **Whole day** |  |  |  |  |  |  |
| Eating occasion frequency (n/day) | 5.9 (5.2; 6.8) | 5.3 (4.8; 6.0) | 6.0 (5.3; 6.7) | 5.8 (5.0; 6.7) | 5.5 (5.0; 6.2) | 5.5 (4.8; 6.0) |
| Meal frequency (n/day) | 4.5 (4.2; 4.8) | 4.4 (4.2; 4.8) | 4.0 (3.8; 4.3) | 4.1 (3.8; 4.5) | 4.0 (3.7; 4.3) | 4.0 (3.7; 4.2) |
| Snack frequency (n/day) | 1.3 (0.8; 2.2) | 0.9 (0.5; 1.4) | 1.8 (1.3; 2.7) | 1.7 (0.8; 2.3) | 1.5 (1.0; 2.2) | 1.4 (1.0; 2.2) |
| **Morning** |  |  |  |  |  |  |
| Eating occasion frequency (n/day) | 1.8 (1.5; 2.1) | 1.8 (1.4; 2.0) | 1.8 (1.7; 2.2) | 1.8 (1.7; 2.2) | 1.7 (1.3; 1.8) | 1.7 (1.3; 1.8) |
| Meal frequency (n/day) | 1.2 (1.1; 1.5) | 1.3 (1.2; 1.5) | 1.3 (1.2; 1.5) | 1.3 (1.2; 1.5) | 1.2 (1.0; 1.3) | 1.2 (1.0; 1.3) |
| Snack frequency (n/day) | 0.4 (0.2; 0.8) | 0.3 (0.1; 0.5) | 0.5 (0.3; 0.8) | 0.5 (0.2; 0.8) | 0.3 (0.2; 0.7) | 0.3 (0.2; 0.5) |
| **Daytime** |  |  |  |  |  |  |
| Eating occasion frequency (n/day) | 2.3 (2.1; 2.6) | 2.2 (2.0; 2.4) | 2.8 (2.5; 3.3) | 2.7 (2.3; 3.2) | 2.7 (2.3; 3.2) | 2.5 (2.2; 3.0) |
| Meal frequency (n/day) | 1.8 (1.7; 2.0) | 1.8 (1.7; 2.0) | 1.8 (1.5; 2.0) | 1.8 (1.7; 2.0) | 1.8 (1.5; 2.0) | 1.7 (1.5; 2.0) |
| Snack frequency (n/day) | 0.5 (0.3; 0.8) | 0.4 (0.2; 0.6) | 1.0 (0.6; 1.5) | 1.0 (0.3; 1.3) | 0.8 (0.5; 1.3) | 0.8 (0.5; 1.2) |
| **Evening** |  |  |  |  |  |  |
| Eating occasion frequency (n/day) | 1.3 (1.1; 1.8) | 1.3 (1.0; 1.5) | 1.2 (1.0; 1.3) | 1.2 (1.0; 1.3) | 1.2 (1.0; 1.3) | 1.2 (1.0; 1.3) |
| Meal frequency (n/day) | 1.1 (1.0, 1.3) | 1.1 (1.0, 1.3) | 1.0 (0.8; 1.0) | 1.0 (0.8; 1.0) | 1.0 (0.8; 1.0) | 1.0 (0.8; 1.0) |
| Snack frequency (n/day) | 0.3 (0.1; 0.5) | 0.1 (0.0; 0.3) | 0.2 (0.0; 0.3) | 0.2 (0.0; 0.3) | 0.2 (0.0; 0.3) | 0.2 (0.0; 0.3) |
| **Nightly eating occassions (n/day)** | 0.7 (0.4; 0.9) | 0.4 (0.2; 0.8) | 0.0 (0.0; 0.0) | 0.0 (0.0; 0.0) | 0.0 (0.0; 0.0) | 0.0 (0.0; 0.0) |
| **Duration of nightly fasting (DNF, min)** | 598 (497; 681) | 683 (603; 741) | 795 (764; 829) | 797 (758; 840) | 801 (767; 837) | 799 (770; 833) |
| **Anthropometric data** |  |  |  |  |  |  |
| Body weight (kg) | 8.3 (7.7; 9.0) | 8.6 (8.0; 9.3) | 15.8 (14.8) | 16.2 (15.1; 18.0) | 22.7 (20.9; 25.1) | 23.4 (21.7; 26.3) |
| BMI-SDS | 0.45 (-0.16; 1.17) | 0.78 (0.17; 1.34) | 0.01 (-0.45; 0.55) | 2.54 (2.10; 2.89) | -0.15 (-0.60; 0.38) | -0.02 (-0.48; 0.69) |
| Overweight status^1^ | 104 (29.6) | 56 (35.5) | 28 (8.0) | 23 (14.6) | 26 (7.4) | 21 (13.3) |
| FMI | 2.91 (2.5; 3.4) | 3.11 (2.58; 3.60) | 2.3 (2.0; 2.8) | 2.53 (2.10; 2.89) | 2.13 (1.78; 2.68) | 2.34 (1.9; 2.9) |
| FFMI | 14.0 (13.5; 14.6) | 14.3 (13.7; 14.8) | 13.1 (12.7; 13.7) | 13.3 (12.7; 13.9) | 13.2 (12.7; 13.7) | 13.3 (12.7; 14.0) |
| **Socioeconomic factors [n (%)]** |  |  |  |  |  |  |
| Maternal overweight^2^ | 102 (29.0) | 53 (33.6) | 107 (30.4) | 52 (32.9) | 124 (35.2) | 56 (35.4) |
| Maternal high educational status^3^ | 277 (78.7) | 109 (69.0) | 279 (79.3) | 105 (66.5) | 283 (80.4) | 107 (67.7) |
| Maternal employment | 71 (20.2) | 24 (15.2) | 164 (46.6) | 67 (42.4) | 259 (73.6) | 102 (64.7) |
| Smoking in Household^4^ | 46 (15.2) | 31 (23.1) | 46 (15.2) | 24 (17.9) | 38 (12.6) | 28 (20.9) |

Values are frequencies (n (%)) or medians (25th; 75th percentile)

EI, energy intake; %E=percentage of energy intake, BMI, body mass index, BMI-SDS = Body mass index – standard deviation scores; DNF = Duration of nightly fasting

^1^Overweight status (BMI-SDS > 90th percentile) was calculated based on the German reference percentiles for children and adolescents by Kromeyer-Hauschild [31]

^2^BMI > 25 kg/m²
^3^≥12 years of schooling

^4^n=50 missings for exclusive breastfed ≥ 4 month; n=24 missings for exclusive breastfed ≤ 4 month

**Additional file 3.** Prospective associations between circadian eating patterns in infancy (0-1 years) with respective eating patterns in pre-school (3-4 years) age of the analysed DONALD sample, stratified by breastfeeding duration (exclusive breastfed ≥ 4 month (n=352) vs. exclusive breastfed < 4 month (n=158))

| **Exclusive breastfed ≥ 4 month** | | | | | | | | | |
| --- | --- | --- | --- | --- | --- | --- | --- | --- | --- |
|  | **Infancy**  **(0-1 years)** | **Pre-school age**  **(3–4 years)** | | | | **Primary-school age**  **(3–4 years)** | | | |
| **Circadian eating pattern** | **Median**  **(25th; 75th percentile)** | **Median**  **(25th; 75th percentile)** | **β** | **P_(FDR)_^1^** | **Median**  **(25th; 75th percentile)** | | **β** | **P_(FDR)_^1^** |  |
| **Eating occasion frequency (n/day)** | 5.9 (5.2; 6.8) | 6.0 (5.3; 6.7) | 0.19 | **<0.01** | 5.5 (5.0; 6.2) | | 0.12 | **<0.01** |  |
| **Duration of nightly fasting (min)** | 598 (497; 681) | 795 (764; 829) | 0.10 | **<0.01** | 801 (767; 837) | | 0.02 | 0.06 |  |
| **Energy from meals (% of TEI)** | 91.8 (86.4; 95.4) | 89.7 (85.6; 92.9) | 0.13 | **<0.01** | 91.6 (88.5; 94.4) | | 0.05 | 0.12 |  |
| **Energy from snacks (% of TEI)** | 10.2 (6.8; 14.6) | 11.8 (8.6; 15.6) | 0.10 | **<0.05** | 10.2 (7.7; 13.4) | | 0.07 | 0.05 |  |
| **Evening Carbohydrate intake [%EI]** | 47.4 (44.6; 51.4) | 47.0 (40.0; 53.1) | -0.05 | 0.66 | 47.8 (42.6; 53.6) | | -0.03 | 0.81 |  |
|  |  |  |  |  |  | |  |  |  |
| **Exclusive breastfed < 4 month** | | | | | | | | |  |
|  | **Infancy**  **(0-1 years)** | **Pre-school age**  **(3–4 years)** | | | **Primary-school age**  **(3–4 years)** | | | |  |
| **Circadian eating pattern** | **Median**  **(25th; 75th percentile)** | **Median**  **(25th; 75th percentile)** | **β** | **P_(FDR)_^1^** | **Median**  **(25th; 75th percentile)** | | **β** | **P_(FDR)_^1^** |  |
| **Eating occasion frequency (n/day)** | 5.3 (4.8; 6.0) | 5.8 (5.0; 6.7) | 0.59 | **<0.01** | 5.5 (4.8; 6.0) | | 0.36 | **<0.01** |  |
| **Duration of nightly fasting (min)** | 683 (603; 741) | 797 (758; 840) | 0.13 | **<0.05** | 799 (770; 833) | | 0.06 | 0.22 |  |
| **Energy from meals (% of TEI)** | 95.1 (92.0; 97.2) | 90.6 (86.2; 94.3) | 0.38 | **<0.01** | 92.0 (88.3; 94.6) | | 0.26 | **<0.05** |  |
| **Energy from snacks (% of TEI)** | 7.0 (4.5; 10.2) | 11.2 (8.0; 14.8) | 0.29 | **<0.05** | 9.9 (7.6; 13.3) | | 0.22 | **<0.05** |  |
| **Evening Carbohydrate intake [%EI]** | 49.4 (46.6; 53.8) | 46.3 (40.6; 52.4) | -0.00 | 0.99 | 48.4 (42.0; 53.9) | | 0.07 | 0.66 |  |
|  |  |  |  |  |  | |  |  |  |

EI = energy intake; TEI=Total daily energy intake; FDR = False discovery rate; DNF = Duration of nightly fasting

^1^P_(FDR)_ refers to P values obtained in linear regression models. Models are adjusted for sex, birthdate, birth weight, maternal high educational status during infancy, maternal employment and weight status during pre-school age. Multiple testing adjustments were performed by controlling the false discovery rate at 5%

**Additional file 4.** Prospective associations between circadian eating patterns in infancy with BMI, FMI, FFMI in primary-school age (6-7 years) of the analysed DONALD sample, stratified by breastfeeding duration (exclusive breastfed ≥ 4 month (n=352) vs. exclusive breastfed < 4 month (n=158))

|  | **BMI-SDS** | | | | **FMI** | | | | **FFMI** | | | |
| --- | --- | --- | --- | --- | --- | --- | --- | --- | --- | --- | --- | --- |
|  | **Exclusive breastfed ≥ 4 month** | | **Exclusive breastfed < 4 month** | | **Exclusive breastfed ≥ 4 month** | | **Exclusive breastfed < 4 month** | | **Exclusive breastfed ≥ 4 month** | | **Exclusive breastfed < 4 month** | |
| **Circadian pattern in infancy** | **β** | P_value_ | **β** | P_value_ | **β** | P_value_ | **β** | P_value_ | **β** | P_value_ | **β** | P_value_ |
| **Eating occasion frequency (n/day)**  Unadjusted Modell  Full adjusted Modell | 0.0065  0.0337 | 0.84  0.31 | -0.0149  0.0097 | 0.85  0.90 | 0.0285  0.0569 | 0.39  0.09 | -0.0764  0.0135 | 0.43  0.89 | 0.0043  0.0239 | 0.90  0.49 | 0.0239  0.0003 | 0.77  0.99 |
| **Duration of nightly fasting (min)**  Unadjusted Modell  Full adjusted Modell | 0.0001  -0.0003 | 0.63  0.37 | 0.0007  0.0003 | 0.33  0.77 | -0.00001  -0.0005 | 0.98  0.18 | 0.0013  0.0006 | 0.10  0.52 | 0.0001  -0.0002 | 0.72  0.50 | 0.0001  -0.0001 | 0.87  0.88 |
| **Energy from meals (% of TEI)**  Unadjusted Modell  Full adjusted Modell | -0.0022  -0.0065 | 0.67  0.22 | 0.0061  0.0036 | 0.70  0.81 | -0.0059  -0.0097 | 0.26  0.06 | 0.0161  0.0067 | 0.41  0.73 | -0.0003  -0.0039 | 0.95  0.48 | -0.0013  0.0004 | 0.94  0.98 |
| **Energy from snacks (% of TEI)**  Unadjusted Modell  Full adjusted Modell | 0.0011  0.0057 | 0.84  0.30 | -0.0079  -0.0028 | 0.62  0.85 | 0.0063  0.0102 | 0.25  0.07 | -0.0187  -0.0072 | 0.33  0.71 | -0.0017  0.0023 | 0.76  0.69 | 0.0002  0.0010 | 0.99  0.95 |
| **Evening Carbohydrate intake [%EI]**  Unadjusted Modell  Full adjusted Modell | -0.0136  -0.0194 | 0.09  0.02***** | 0.0329  0.0215 | **0.01***  0.10 | -0.0157  -0.0148 | 0.06  0.07 | 0.0336  0.0267 | **0.03***  0.10 | -0.0055  -0.0171 | 0.52  **0.04*** | 0.0310  0.0155 | **0.02***  0.24 |
|  |  |  |  |  |  |  |  |  |  |  |  |  |

BMI-SDS = Body mass index – standard deviation scores; FMI = fat mass index; FFMI= Fat free mass index; TEI = Total daily energy intake

P refers to P values obtained in linear regression models. Fully adjusted model adjusted for sex, birthdate, birth weight, maternal high educational status during infancy, maternal employment and weight status during pre- or primary-school age and for total daily energy intake during infancy.

*Significance p < 0.05 did not remain after the adjustment for multiple testing according to the false discovery rate.
